# Pattern formation in drying blood drops

**DOI:** 10.1098/rsta.2020.0391

**Published:** 2021-08-09

**Authors:** Michael. J. Hertaeg, Rico F. Tabor, Alexander F. Routh, Gil Garnier

**Affiliations:** ^1^ BioPRIA and Department of Chemical Engineering, Monash University, Clayton, Victoria 3800, Australia; ^2^ School of Chemistry, Monash University, Clayton, Victoria 3800, Australia; ^3^ Department of Chemical Engineering and Biotechnology, University of Cambridge, Cambridge, Cambridgeshire CB3 0AS, UK

**Keywords:** drying, coffee ring, blood, droplet drying, diagnostics

## Abstract

Patterns in dried droplets are commonly observed as rings left after spills of dirty water or coffee have evaporated. Patterns are also seen in dried blood droplets and the patterns have been shown to differ from patients afflicted with different medical conditions. This has been proposed as the basis for a new generation of low-cost blood diagnostics. Before these diagnostics can be widely used, the underlying mechanisms leading to pattern formation in these systems must be understood. We analyse the height profile and appearance of dispersions prepared with red blood cells (RBCs) from healthy donors. The red cell concentrations and diluent were varied and compared with simple polystyrene particle systems to identify the dominant mechanistic variables. Typically, a high concentration of non-volatile components suppresses ring formation. However, RBC suspensions display a greater volume of edge deposition when the red cell concentration is higher. This discrepancy is caused by the consolidation front halting during drying for most blood suspensions. This prevents the standard horizontal drying mechanism and leads to two clearly defined regions in final crack patterns and height profile.

This article is part of a discussion meeting issue ‘A cracking approach to inventing new tough materials: fracture stranger than friction’.

## 1. Introduction

Blood droplet analysis has been used in forensics for many years [1]. It is only recently that its medical applications have received attention, where the patterns produced in dried blood are indicative of several important medical conditions [2,3]. These include carcinoma [4], anaemia, hyperlipemeia [5], thalasemia, jaundice [6] and many more [7]. These findings indicate the potential for a new generation of low-cost point of care diagnostics where all that is required is a droplet of patient blood and a standard surface. Although these techniques are promising, a better understanding of the fundamentals behind pattern formation in these systems is required before any robust tests can be efficiently designed, and results relied upon for diagnosis. This is because the mechanisms that lead to pattern formation in drying droplets of blood are poorly understood.

**Figure 1.**
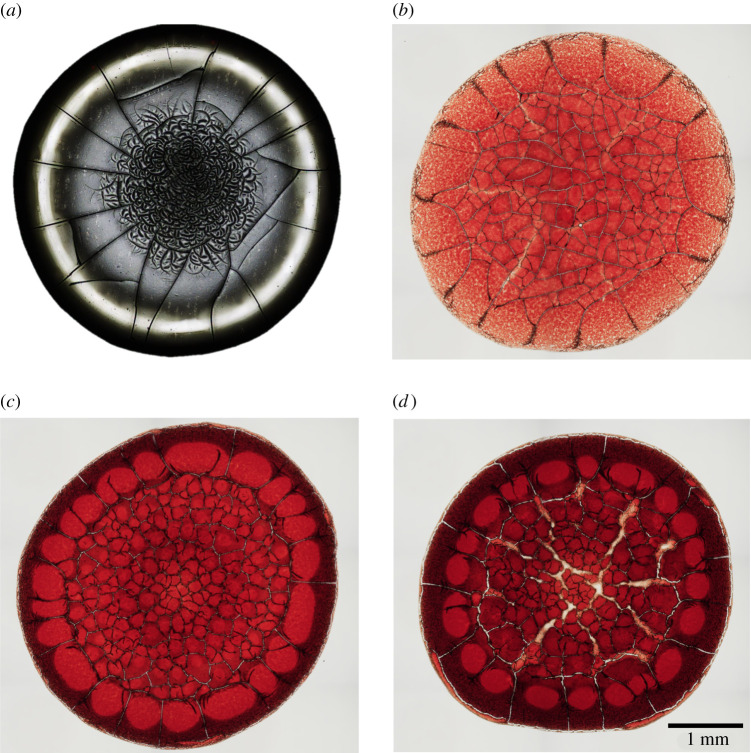
Images of droplets dried on untreated glass. Plasma with different concentrations of RBCs: (*a*) 0 vol.%, (*b*) 15 vol.%, (*c*) 30 vol.%, (*d*) 45 vol.%. Scale bar applies to all images. (Online version in colour.)

Much of the previous research on the analysis of dried blood droplets has focused on describing the appearance of cracking patterns [8–11]. These are easily seen by eye and therefore have potential to be used for diagnostics. The cracking patterns of blood are observed to be strongly influenced by contact angle and spreading dynamics [12] as well as relative humidity (RH) due to changes in evaporation rate [8,13]. Crack appearance was demonstrated to be affected by droplet size and thickness [5,10]. The appearance of the patterns formed by dried blood droplets with several different initial concentrations of red blood cells (RBCs) in plasma are shown in figure 1. Crack initiation and propagation in drying dispersions is a complex yet reproducible process that is highly dependent on the properties of the particle system [14]. This is why pattern formation can differentiate between small variations caused by illness. The concept behind these diagnostics is to visually analyse crack patterns in dried droplets deposited on a model surface. Any change in RBC concentration, shape, rigidity or plasma surface tension will alter the dynamics and final appearance, thereby relating crack patterns to pathology. There are many mathematical models that are used to predict cracking patterns in a variety of drying systems [15–19], although complications in blood systems make their application difficult. One such complication is the redistribution of components that occurs in the early stages of drying, which is responsible for the ring-like profiles that develop in most circumstances [20]. This process has been studied extensively in recent years, evidenced by the quantity of recent books and reviews on the topic [18,21–30].

**Figure 2.**
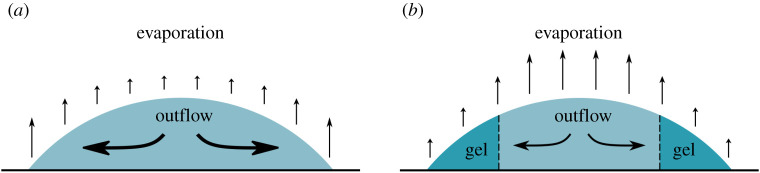
Schematic showing the drying of a droplet of blood. (*a*) Early times, similar to process in pure fluids. (*b*) Mid-late times showing the formation of a consolidated/gelled region with no fluid flow and a reduced evaporation. (Online version in colour.)

The drying behaviour of a droplet of whole blood and the relationship with pathology was first reported by Brutin *et al.* [5]. This process is sketched in figure 2. By monitoring weight change during drying two distinct regions were identified. The first is a diffusion dominated region characterized by a rapid rate of mass loss. In this region, the evaporation rate is similar to the evaporation of droplets of pure solvent [31]. In the second stage, evaporation of the remaining fluid is limited by transport through the gellified matrix that forms once a critical volume fraction is reached. There is a short transition zone as gelling occurs first on the droplet’s edge and then propagates inwards [10]. Sobac and Brutin used a diffusive model to predict the transition point between these two regions [31]. They later used a similar model to predict the rate of evaporation in the first region by fitting evaporative area to the experimentally determined area of the central fluid region [10]. This demonstrated that evaporative processes in blood drops are governed by the relative effects of convection, diffusion, and gelation in the liquid phase.

In this work, the drying process of droplets varying in RBC and protein content are analysed. The motivation for this work is to elucidate the dominant processes that govern pattern formation in blood droplets. The time evolutions of RBC suspensions are compared with simple colloidal particles. Dried profiles are compared with RBC suspensions in bovine serum albumin (BSA), phosphate buffered saline (PBS) and blood plasma solutions. Experiments are performed on smooth glass surfaces with plasma treatment and without, providing a variation in initial contact angles. Note that we have used two types of plasma in this study: the radiation used to treat substrates and the liquid component of blood.

## 2. Methods

Model RBC suspensions were made using whole blood provided by the Red Cross Australia, with ethics approval from the Australian Blood Service Human Research Ethics Committee (BSHREC) and the Monash University Human Research Ethics Committee (MUHREC). EDTA was used as an anticoagulant. RBCs were washed three times by centrifugation, removing the supernatant, and were then re-suspended in PBS. The pellet left after centrifugation was assumed to be 100 vol.% cells, which was combined with the selected solution to obtain the required concentration. Three different solutions were used: (1) Plasma, which was collected from the same blood sample by removing the supernatant after the first washing step; (2) PBS, which was purchased from Sigma and made up with water purified with a Millipore Milli-Q system; (3) BSA, which was diluted in PBS from 30% (wt/v) solutions from BioCSL. When plasma was used as a diluent, dilution of RBCs was performed with plasma and cells from the same donor to avoid possible immune reactions. Six millilitre droplets of suspensions were deposited onto glass microscope slides (Westlab, Ballart, Australia) with an Eppendorf automatic pipette and left to dry at 23°C, 50% RH. Wettability of glass slides was altered by treatment with a PDC-002-HP Harrik Plasma, plasma cleaner for 3 min using the medium setting. The effect of surface treatment was quantified by measuring the contact angle of tested fluids. Contact angle measurements were performed with an OCA35 DataPhysics contact angle instrument within one minute of depositing the droplet. The presented contact angles are the mean of at least four measurements using cells from at least two different donors. Height profile scans were measured using an Olympus LEXT OLS5000 laser confocal microscope. Four replicates of each test were performed, comprising two results from two different donors. Profilometry figures show all of these measurements together. Polystyrene beads with a 6–8 μm nominal diameter were purchased from PROSCITECH and were concentrated to 15% by volume by centrifuging at 2500 rcf for 3.5 min with subsequent removal of the supernatant. Deuterium oxide from Sigma-Aldrich was mixed with solutions to density match the particles and the fluid. This was performed by using an initial solution concentration of 50 vol.% D_2_O–50 vol.% H_2_O. The concentration of D_2_O was then incrementally increased by centrifuging and replacing a small volume of the supernatant with the same volume of D_2_O. This process was repeated until a 3.5 min centrifuge at 2500 rcf did not cause phase separation. Due to impurities in the D_2_O source, the final concentration varied slightly. However, the concentration of the solutions was approximately 65 vol.% D_2_O. Care was taken to undertake experiments in still air; however, slight directionality was seen in many tests due to a sensitivity to air circulation present in the laboratory environment.

## 3. Results

Six microlitre droplets varying in RBC concentration and diluent (plasma, PBS and BSA solutions) were deposited on smooth glass surfaces with and without plasma treatment. Dried droplet profiles and crack pattern were measured as a function of drying time under constant environmental conditions (50% RH and 23°C). Six microlitre droplets were chosen so that features were large enough to be observed by eye while keeping the Bond number low. Using the droplet height (0.86 mm) as the characteristic length scale, 1050 kg m^−3^ and 57 mN m^−1^ for the density [32] and surface tension [33] of whole blood, respectively, the Bond number is calculated to be 0.13. Due to the high contact angle and lower surface tension of whole blood, this provides the highest Bond number out of all tested droplets.

**Figure 3.**
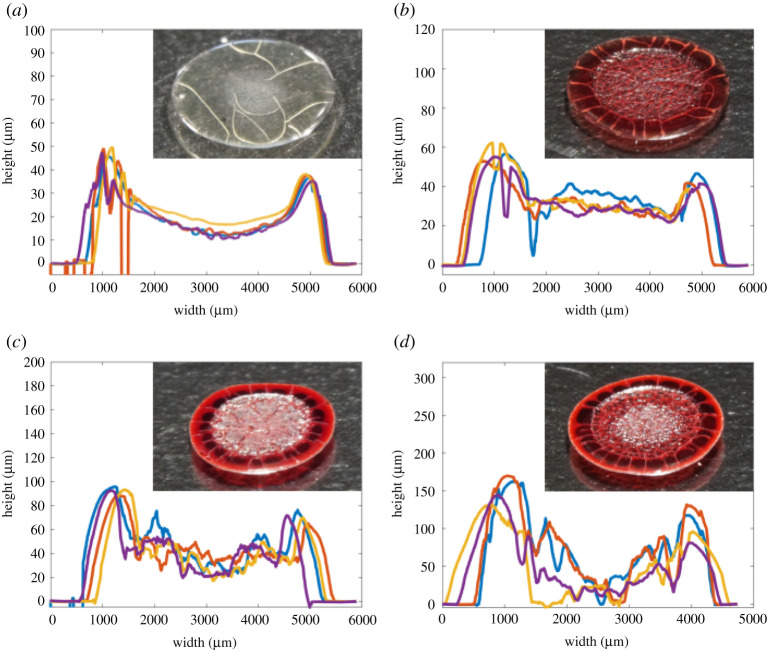
Profilometry centerline scans of dried 6 μl droplets of RBCs suspended in plasma on untreated glass. Insets show representative images of dried deposits. Effect of RBC concentration in plasma: (*a*) 0 vol.%, (*b*) 15 vol.%, (*c*) 30 vol.% and (*d*) 45 vol.%. (Online version in colour.)

**Figure 4.**
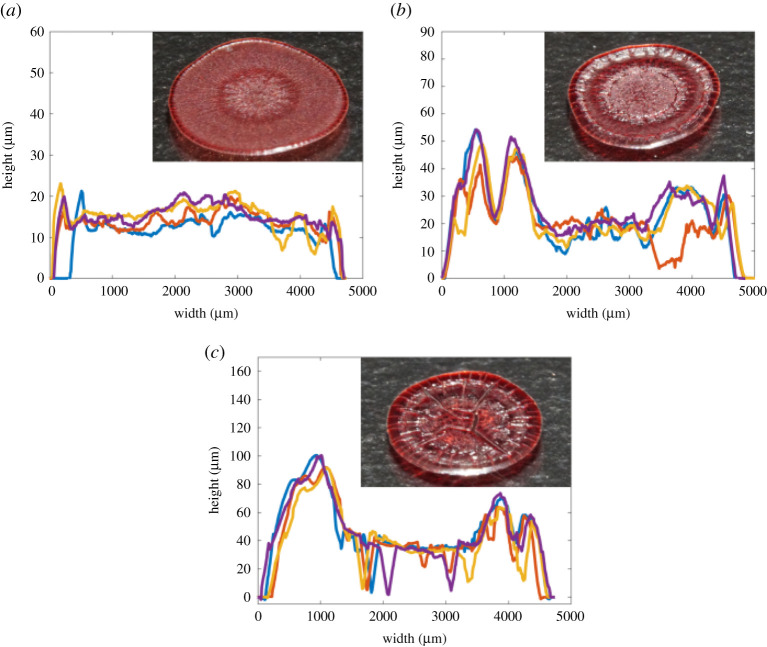
Profilometry centerline scans of dried 6 μl droplets of RBCs suspended in PBS solutions on untreated glass. Insets show representative images of dried deposits. Effect of RBC concentration in PBS: (*a*) 15 vol.%, (*b*) 30 vol.% and (*c*) 45 vol.%. (Online version in colour.)

**Figure 5.**
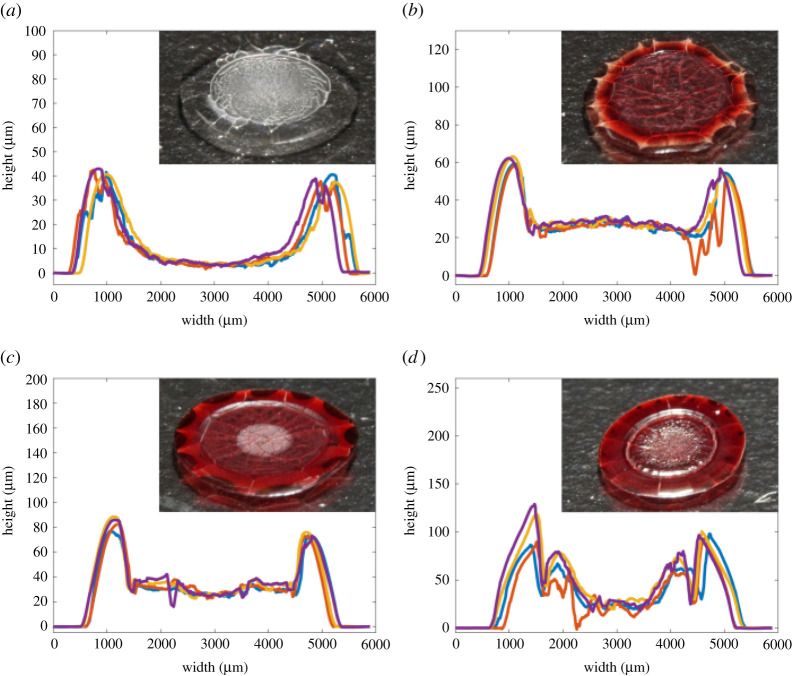
Profilometry centerline scans of dried 6 μl droplets of RBCs suspended in BSA solutions on untreated glass. Insets show representative images of dried deposits. Effect of RBC concentration in BSA: (*a*) 0 vol.%, (*b*) 15 vol.%, (*c*) 30 vol.% and (*d*) 45 vol.%. (Online version in colour.)

Figure 3 shows the centre line profilometry scans of dried droplets of RBCs combined with human plasma, on untreated glass at four different concentrations of RBC: 0%, 15%, 30% and 45% by volume. 45 vol.% is the approximate concentration of red cells in whole human blood [5]. These figures show high reproducibility and a distinct relationship with concentration. A ring-like profile, or coffee ring, is always observed, with a greater volume of edge deposition present at high RBC concentrations. Figure 4 shows the profilometry measurements of RBCs suspended in PBS solutions under the same conditions. Despite having the same ionic strength and pH as plasma, dried profiles of RBCs suspended in PBS solutions demonstrated less defined ring profiles and fewer large cracks. Also, the lower cell concentrations of PBS solutions displayed almost uniform final height profiles. RBC suspensions in BSA solutions were also tested (figure 5) on untreated glass. An 83 g l^−1^ BSA solution in PBS was chosen as this showed plasma similar properties in another study [34]. Suspensions with BSA produced profiles similar to those with plasma, although results were more reproducible and slightly fewer large cracks were observed. All dried droplet profiles are reproducible and characteristic of the RBC concentration and the type of diluent.

In all tests, a higher initial concentration of RBCs resulted in a greater volume of edge deposition. This is opposite to predictions and experiments with simple dispersions [35,36], where a high initial concentration was observed to suppress ring deposits in simple colloid suspensions. When RBCs are suspended in a protein solution the relationship with initial concentration is less defined because protein solutions with no cells produce ring structures. However, there is still a clear trend between degree of edge accumulation and initial RBC concentration.

**Figure 6.**
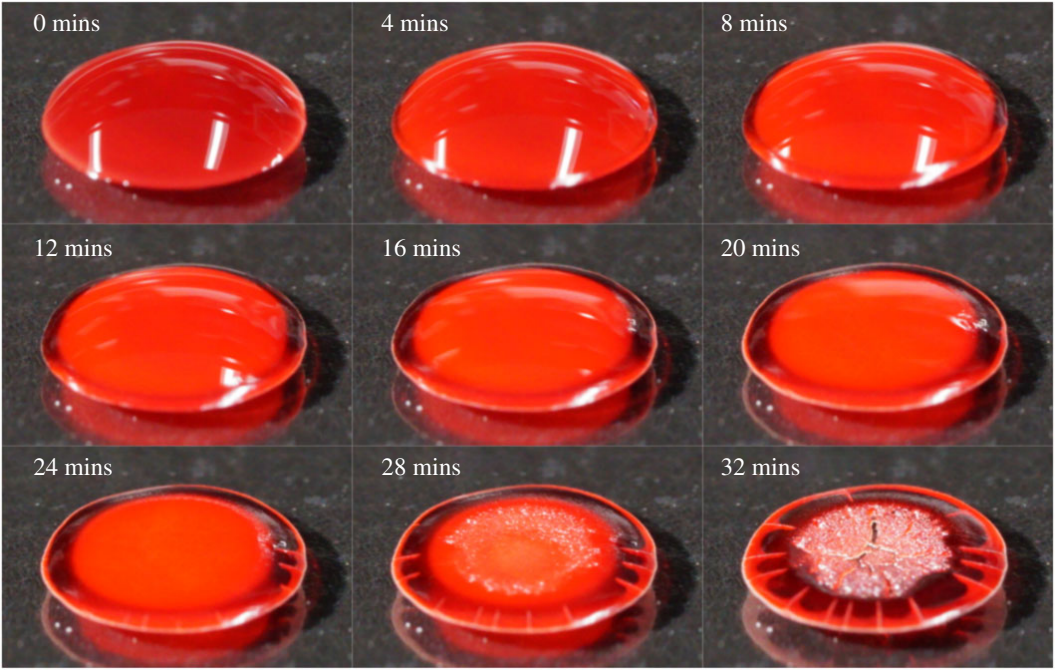
Photographs showing the drying of a droplet of whole blood on untreated glass. The approximate diameter of the droplet was 4 mm. (Online version in colour.)

Figure 6 shows a series of photographs taken at 4 min intervals that display the time evolution of droplets (6 μl) of whole blood. Images are displayed until no visual difference was observed between subsequent images. Similar recordings for pure plasma, RBCs in PBS and a polystyrene suspension (15% by volume) were captured and are shown in electronic supplementary material. For all systems, a ‘compaction’ front [10] is identifiable within a short time at the outer edge, which propagates inwards. For polystyrene and plasma, this front continues until it reaches the centre. The solid front reaching the centre is marked for polystyrene by a sudden colour change and for plasma by crack initiation. Both events imply complete consolidation of particles [37]. For whole blood and concentrated RBC suspensions in PBS solutions, the front halts before reaching the centre followed by a near simultaneous drying of the remaining fluid region [5,10].

The effect of contact angle was investigated. Table 1 displays the contact angle of the suspensions used on two different surfaces: untreated and treated glass. Both plasma and PBS suspensions display similar contact angles. However, results with both diluents show a decreasing contact angle with decreasing RBC concentration.

**Table 1.** Contact angle of RBC suspensions in plasma and PBS over treated glass surfaces. The quoted error is the standard deviation arising from at least four measurement from two different donors.

vol.% of RBC	RBCs in plasma untreated glass	RBCs in PBS untreated glass	RBCs in plasma plasma-treated glass	RBCs in PBS plasma-treated glass
0%	38.2 ± 9.9	—	8.4 ± 2.1	—
15%	37.8 ± 10.6	31.0 ± 4.9	7.9 ± 1.1	7.0 ± 1.2
30%	33.8 ± 6.8	35.7 ± 5.0	8.4 ± 1.0	6.4 ± 0.7
45%	45.6 ± 7.6	38.7 ± 6.9	9.1 ± 0.9	7.5 ± 0.8

**Figure 7.**
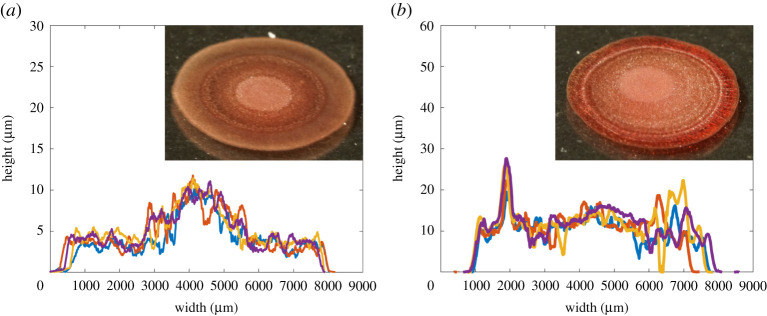
Profilometry scans and photographs of dried droplets of two different concentrations of RBCs suspended in PBS solutions on treated glass, controlling contact angle. (*a*) 15 vol.% and (*b*) 30 vol.%. (Online version in colour.)

Profilometry results showed that the effect of contact angle was minimal, with many deposits being similar in shape to their higher contact angle counterparts despite being wider and thinner. A notable exception to this is a transition that occurs at low contact angles and low RBC concentrations in PBS suspensions, where a central deposit is observed. Profilometry recordings and images of deposits around this transition are displayed in figure 7. These recordings can be compared to figure 4*a,b* as they are the same solutions dried at different contact angles.

## 4. Discussion

### (a) Effect of proteins

Comparison of figures 3–5 highlights the large impact that plasma has on the dried profiles of droplets, indicating the dominant role of proteins. The ring profile produced by a neat plasma solution on glass demonstrates that plasma by itself can induce ring formations because of the presence of proteins [3]. Transport and deposition of proteins to the edge of the drop occurs by convection [38–41]. Just as for suspensions of solid particles there is a critical volume fraction at which the fluid solidifies and a front of solidified material initiates at the edge of the droplet which propagates inwards. However, this mechanism is governed by the gelling or crystallization of protein and salt molecules rather than physical packing as occurs with latex suspensions.

Drying of multicomponent droplets has been shown to preferentially transport smaller elements to the edge [42]. This is because the smaller components can flow between larger particles, and therefore be transported past the solid front. This is also true for proteins, as these macromolecules are dissolved in the liquid phase and transport freely to the edge of the drying droplet. The preferential deposition of proteins at the edge not only slows evaporation in this region but also hinders the development of interparticle menisci, which are responsible for capillary pressure generation. This protein induced shift in dynamics is likely to be responsible for the observed dependence of dried droplet morphology with protein content.

Initially, tests with RBCs in PBS have little to no proteins in solution, although as drying proceeds the solution becomes increasingly concentrated. This causes water loss through osmosis leading to significant deformation (crenation) of RBC membranes and a percentage of cells will rupture due to mechanical stresses (haemolysis) [43,44]. This releases haemoglobin and other proteins into the bulk solution, which behave similarly to the plasma proteins. Through this mechanism, proteins are introduced to the solution in the later stages of drying, which explains similarities between the PBS and plasma suspensions at high RBC concentrations. Another mechanism which is ignored in this study is blood clotting due to fibrinogen and the cascade of blood-co-factors. Fibrinogen would not only tighten RBC packing but also affect the mechanical properties of RBC films, therefore possibly influencing the crack formation pattern of dried blood. The clotting mechanism is not relevant to the experiments reported here because of the addition of EDTA anticoagulant at donation.

### (b) Mechanism

For plasma and polystyrene suspensions (electronic supplementary material), the front continues to propagate until the centre. This complete consolidation is followed by continued water loss and cracking or decolouration within the whole droplet. Before this occurs, there is a significant amount of fluid in the outer region. For blood (figure 6), the consolidation front slows down and effectively stops, demonstrating a different drying mechanism to polystyrene latex. Cracks are observed in the outer region [10], which implies a significant evaporation induced pressure drop along the consolidated region, before the compaction front reaches the centre. This was also implied by Sobac *et al.* [10] who demonstrated that evaporation in the outer region of blood droplets is severely diminished.

The motion of the front and the amount of fluid present in different regions is relevant because it indicates the internal flow that is occurring. In simple colloidal systems, such as the polystyrene suspension presented here, evaporation from the consolidated region causes the fluid to flow outward through the packed bed resulting in a negative capillary pressure. The outward flow of fluid is balanced by a flow in the fluid inner region [21]. This transports particles to the consolidation front, which pack and cause the propagation of the front and the typically observed ring profiles. The flow described above continues until either the consolidation front reaches the centre of the drop or the maximum capillary pressure of the packed particle system is reached at the droplet edge [37]. When this occurs, the outflow from the inner region decreases, slowing the front progression. As lateral flow is reduced, evaporation can become dominant in the outer region, drying the remaining fluid.

The polystyrene and plasma tests presented in the electronic supplementary material resulted in constant progression of the consolidation front and then sudden drying after the front reached the centre. This implies that the outer region remains saturated with fluid and an outflow continues until the very late stages of drying. For blood, the slow-down in front progression and cracking in the outer region implies that the outer region de-wets very early and there is very little outflow after this point.

The difference in drying dynamics in blood droplets causes the observed shift in final profile. After drying in the outer region, further evaporation causes the height to decrease in the inner region but does not affect the position of the front. This makes the shape of the fluid interface invert and form the concave shape seen in dried morphologies. As the inner region is drying uniformly, the local concentration is similar, producing the observed instantaneous gelation once the critical volume fraction is reached.

This is a very different process of ring formation from that observed in simple colloid systems, which explains the contrary relationship with initial concentration. Our proposed mechanism also explains the abrupt change in both cracking patterns and height profile seen between the inner and outer regions in dried blood droplets. The outer corona has regularly spaced radial cracks [5], typical of horizontal drying fronts [37]. The cracks in the inner region are more irregular, leading to a pattern called ‘mud cracks’ that are often observed in one-dimensional vertical evaporation [45]. These two regions were also observed by profilometry. The higher concentration suspensions in figures 3–5 show a discontinuity at the point where the front halted and a higher roughness in the central region. Although two regions can be identified in the image of plasma cracks in figure 1*a*, the timing of their formation presented in the electronic supplementary material identifies a different mechanism to blood as the central random cracks propagate outwards, after the solid front reaches the centre of the drop.

To investigate this process further, an analytical model was developed using equation (4.1) which was used by previous researchers [35,36,46,47] to represent dimensionless droplet height (*h*) as a function of dimensionless time (*t*) and radial position (*r*). It is derived assuming lubrication theory and that pressure is solely a function of local curvature. This is combined with a transport equation for volume fraction (*ϕ*) to determine the distribution of the non-volatile component (equation (4.2)). Solidification is implemented by specifying a critical volume fraction (*ϕ*_max_) where the fluid will solidify and undergo no further changes in height. In this way, previous researchers have predicted the front progression and the final height profile upon drying for a range of systems.

We use the droplet radius *R* to scale horizontal distances, and the initial height of the droplet *H* to scale vertical distances. The factor $R\dot{E}/H$ is used to scale horizontal velocities and vertical velocities are scaled by $\dot{E}$, where $\dot{E}$ is the rate of evaporation which is assumed to be constant. Pressure is scaled by $\mu \dot{E}R^{2}/H^{3}$ and time is scaled by $H/\dot{E}$. With these substitutions the equation can be represented in the form presented here, where the term $H^{4}\gamma /3\mu \dot{E}R^{4}$ is analogous to the inverse of the capillary number.

4.1 $$1+\frac{\partial h}{\partial t}+\frac{H^{4}\gamma }{3\mu \dot{E}R^{4}}\frac{1}{r}\frac{\partial }{\partial r}\left[h^{3}r\frac{\partial }{\partial r}\left(\frac{1}{r}\frac{\partial }{\partial r}\left(r\frac{\partial h}{\partial r}\right)\right)\right]=0$$

and

4.2 $$\frac{\partial (rh\phi \tilde{V_{r}})}{\partial r}+\frac{\partial (h\phi r)}{\partial t}=0.$$

Using the same non-dimensional constants the height averaged velocity is expressed as

4.3 $$\tilde{V_{r}}=\frac{H^{4}\gamma }{3\mu \dot{E}R^{4}}h^{2}\left[\frac{\partial }{\partial r}\left(\frac{1}{r}\frac{\partial }{\partial r}(r\frac{\partial h}{\partial r})\right)\right].$$

The example we consider is a drying droplet of blood with an initial height profile of a spherical cap with an initially uniform volume fraction (*ϕ*_0_) and zero flow into the consolidated region. In a droplet of non-deformable particles, outflow into the consolidated region is the primary cause of fluid flow. Blood droplet drying occurs in the limit of surface tension dominating viscous forces. This is formally shown by calculating the value of the dimensionless term in equation (4.1). Using the conservative case of a 6 μ l whole blood drop on untreated glass *H* = 0.86 × 10^−3^ m, *R* = 2.9 × 10^−3^ m, μ = 4.0 × 10^−3^ Pa s , *γ* = 50 × 10^−3^ N m^−1^ and $\dot{E}=3.5\times 10^{-8}\;\text{m}\;{\text{s}}^{-1}$. Values of μ [48], *γ* [49] and $\dot{E}$ [5,50] are taken from literature. This leads to a value of $H^{4}\gamma /3\mu \dot{E}R^{4}$ of 9.2 × 10^5^ demonstrating the dominance of surface tension. This allows the simplification of equation (4.1),

4.4 $$\frac{\partial }{\partial r}\left[h^{3}r\frac{\partial }{\partial r}\left(\frac{1}{r}\frac{\partial }{\partial r}\left(r\frac{\partial h}{\partial r}\right)\right)\right]=0,$$

which is satisfied with a quadratic height profile. Within the lubrication approximation this is equivalent to maintaining a spherical cap which is expected in surface tension dominated flows.

If this quadratic height profile for *h* is used in equation (4.3), then velocity can be calculated to be zero at all points.

Using an initial quadratic profile *h*_0_ = 1 − *r*^2^ and the assumption of zero velocity, height can be calculated as a simple function of the initial height and the amount of evaporation that has occurred (equation (4.5)). The zero velocity assumption also allows a simple expression for volume fraction (equation (4.6)).

4.5 $$h=h_{0}-t$$

and

4.6 $$\phi =\frac{\phi_{0}h_{0}}{h_{0}-t}.$$

When a critical volume fraction is reached the fluid will solidify. Combining equations (4.5) and (4.6) gives the position of the front as a function of time.

4.7 $$r_{\text{front}}=\sqrt{1-\frac{t}{A}},$$

where

4.8 $$A=\frac{\phi_{\text{max}}-\phi_{0}}{\phi_{\text{max}}}.$$

From this, the height profile of the drop can be calculated as a function of time and position

4.9 $$h(r,t)=\left\{ \begin{array}{ll} 1-r^{2}-t, & r<\sqrt{1-\frac{t}{A}}\\ \frac{\phi_{0}}{\phi_{\text{max}}}(1-r^{2}), & r>\sqrt{1-\frac{t}{A}}. \end{array}\right.$$

This analysis shows that front progression can occur when there is no outflow. However, this produces a front velocity that increases with time (equation (4.7)) and the final thickness is a scaled version of the initial quadratic height profile. For formation of a ring in the final dried structure an outflow is required. As dried blood droplets display ring formation, we conclude that an outflow occurs at least initially. The cessation of front progression, as seen when drying whole blood and RBC in plasma, is due to deformation of the RBC in the consolidated region, caused by the capillary pressure of solvent flowing towards the droplet edge. Once the RBC have completely deformed into a structure with volume fraction unity, there is no further flow of solvent through the consolidated region and the droplet reverts to the one-dimensional drying outlined above. Curvello *et al.* [51] showed agglutinated RBCs to adopt a close-packed hexagonal shape, demonstrating that red cells can deform to form a continuous material. Therefore, we postulate that the thickness of the consolidated region when it ceases inward movement is a direct measure of the RBC rigidity.

An alternate mechanism leading to ring formations in the absence of an outflow into the consolidated region relies on an edge enhanced evaporation. This was analysed by Tarasevich *et al.* [52] and predicted ring formation behaviour similar to that seen in simple particles. Edge enhanced evaporation is assumed by many droplet researchers and has been demonstrated in still air [20,53,54] due to mass transfer limited evaporation. However, the surrounding airflow required to achieve this is so low that it would be unlikely in laboratory environments without significant control measures [14]. Therefore, a uniform evaporation profile over the fluid region is often assumed.

### (c) Front slow down

Slow down and halting of the compaction front in drying blood droplets has a large effect on the final droplet appearance. However, due to the complexity of blood, there are several complications surrounding this observation. As RBCs are highly deformable and originally biconcave in shape, they will pack efficiently, causing the packing fraction in the consolidated region to be much closer to unity than equivalent systems of hard spherical particles. This causes two primary differences when compared to simple particles: first, the increased resistance to solvent flow caused by greater particle volume fraction hinders outward flow and consequently decreases the rate of accumulation at the front [55]. Second, the increased packing efficiency means that a greater bulk flow is required to produce the same volume of consolidated cells. Dehydration of the cells in this region decreases their average size, increasing their packing fraction and also acts as a source of solvent, negating the flow from the fluid region. All of these effects will lead to slower front progression when compared with rigid spherical particles. The halting of the front could also be influenced by a layer of gelled proteins at the air interface, restricting evaporation. Proteins will be preferentially transported to the droplet’s edge, therefore also disturbing the formation of interparticle menisci.

Another hypothesis that could cause the slowing of the front is a gelation process occurring in the fluid region. This could occur well before a change is visually observable and would create a viscous, poroelastic matrix in the central region which restricts flow into the consolidated region. Despite its simplicity, this mechanism is unlikely to apply as the fluid region is seen to retain fluid-like properties until the very late stages of drying. This was demonstrated by the unaltered evaporation occurring in the fluid region [10] and the observation that red cells readily flow into paper strips dipped into the central region after the front has halted.

### (d) Effect of surface wettability (contact angle)

The effect of contact angle on dried deposits of blood drops was studied by Sobac *et al.* [10]. Droplets with a contact angle of 92° produced a very different dried profile from those with a contact angle of 15°. For the high contact angle cases, the process is dominated by skin formation followed by buckling, in a similar process to that observed in suspensions with low particle diffusivity [56]. For this study, the profiles were compared between smaller variations in contact angle. The minimal variation in contact angle for blood droplets further identifies the different drying mechanism of RBC suspensions when compared to simple dispersions.

It is not clear what mechanism leads to the central deposit observed for low contact angle droplets in figure 7. However, central deposits are observed in other colloidal droplet systems and correlated with Marangoni flows arising from temperature gradients over the drop’s surface [57]. The central deposit is reproducibly observable by eye, indicating potential for such transitions to be used in the development of low-cost blood diagnostics. We postulate that the critical contact angle under which a central deposit is observed, can indicate the volume fraction of red cells in a sample.

## 5. Conclusion

Here, height profiles of a series of RBC suspensions dried on smooth treated glass slides were measured and their relationship with initial cell concentration, protein content, diluent and contact angle was investigated. Time evolutions are compared with simple latex suspensions. The primary difference between these in droplets of RBC and that of simple colloidal suspensions is halting of the compaction front. This occurs in RBC suspensions shortly after drying has initiated. Possible causes include increased friction due to better packing efficiency, and disturbance of interparticle menisci at the air-dispersion interface. This study has neglected the biochemical cascade reactions involving fibrinogen and the coagulation co-factors. Further experimental and theoretical analysis is required to more completely elucidate this mechanism. The halting of the front in RBC suspensions causes a different mechanism of ring formation based on the drying and inversion of the central fluid region. This is very different to the typical ring formation mechanism in simple suspensions of non-deformable particles where compaction front propagation continues until the centre of the droplet is reached. Altering the substrate/dispersion contact angle had negligible effects on the dried profile obtained, except for RBC suspensions in PBS solutions at low initial concentration and contact angle, where a central platform was observed.

## Data accessibility

Electronic supplementary material figures showing the drying of plasma, RBC+PBS and polystyrene bead systems and data from profilometry centerline scans are shown in electronic supplementary material.
